# Pitfalls of Mitochondrial Redox Signaling Research

**DOI:** 10.3390/antiox12091696

**Published:** 2023-08-31

**Authors:** Petr Ježek

**Affiliations:** Department of Mitochondrial Physiology, No. 75, Institute of Physiology of the Czech Academy of Sciences, Vídeňská 1083, 14220 Prague, Czech Republic; jezek@biomed.cas.cz; Tel.: +420-296442760

**Keywords:** redox signaling from mitochondria, matrix H_2_O_2_ release, H_2_O_2_ release into intracristal space, cytosolic H_2_O_2_ release, redox buffers, peroxiredoxins, redox-sensitive probes, MnSOD

## Abstract

Redox signaling from mitochondria (mt) to the cytosol and plasma membrane (PM) has been scarcely reported, such as in the case of hypoxic cell adaptation or (2-oxo-) 2-keto-isocaproate (KIC) β-like-oxidation stimulating insulin secretion in pancreatic β-cells. Mutual redox state influence between mitochondrial major compartments, the matrix and the intracristal space, and the cytosol is therefore derived theoretically in this article to predict possible conditions, when mt-to-cytosol and mt-to-PM signals may occur, as well as conditions in which the cytosolic redox signaling is not overwhelmed by the mitochondrial antioxidant capacity. Possible peroxiredoxin 3 participation in mt-to-cytosol redox signaling is discussed, as well as another specific case, whereby mitochondrial superoxide release is diminished, whereas the matrix MnSOD is activated. As a result, the enhanced conversion to H_2_O_2_ allows H_2_O_2_ diffusion into the cytosol, where it could be a predominant component of the H_2_O_2_ release. In both of these ways, mt-to-cytosol and mt-to-PM signals may be realized. Finally, the use of redox-sensitive probes is discussed, which disturb redox equilibria, and hence add a surplus redox-buffering to the compartment, where they are localized. Specifically, when attempts to quantify net H_2_O_2_ fluxes are to be made, this should be taken into account.

## 1. Introduction

Redox homeostasis in mitochondrial (mt) compartments is interrelated with redox homeostasis in cytosol and plasma membrane (PM). In many cell types, specifically, where mitochondrial volume accounts for a large fraction of the cell volume, the mitochondrial formation of superoxide and downstream reactive oxygen species (ROS) significantly contributes to the overall cell ROS sources and the established redox state. Balance between pro-oxidant and antioxidant processes determines the redox state within the particular cell compartments and/or organelles. When such balance is shifted so that ROS sources prevail or antioxidant mechanisms are diminished, oxidative stress arises above a certain threshold, distinct in different cell types. However, below such oxidative stress thresholds, transient increases in ROS exist, typically H_2_O_2_ elevations, that represent redox signaling. Redox signals can act within the same single compartment or between distinct compartments. In this review article, the acute redox signaling from mitochondria to the cytosol is discussed, i.e., within a time frame of minutes or when only post-translational modifications may be effective, but not transcriptome reprogramming. The validated examples are reviewed, and theoretical relations are predicted. A specific case is pointed out, when directions of redox/oxidation state have opposite trends, i.e., when the matrix superoxide release diminishes, whereas the cytosolic H_2_O_2_ release is elevated. Long-term redox regulations are not discussed.

It should be stated that reports of acute redox signaling from mitochondria are rather scarce. The reader can also refer to excellent reviews published elsewhere [1,2,3,4]. One of the validated cases of redox signals from mitochondria is concerned with the initiation of hypoxia-induced factor- (HIF)-mediated transcriptome reprogramming [5,6]. Other redox-sensitive gene-regulatory processes, spanning various time scales, have also been reported, such as in progression through the S-phase of the cell cycle [7] and the regulation of quiescence, activation, proliferation, and differentiation of stem cells [8]. A class of mt redox burst processes arises from the succinate accumulation and its sudden subsequent termination leading to the reverse electron transfer (RET) and very intensive ROS bursts (pathological, upon hypoxia/reoxygenation, i.e., ischemia/reperfusion [9]) or less-intensive ROS bursts, representing physiological redox signaling [10,11].

## 2. Pancreatic β-Cells as Exemplar Milieu for Mitochondrial and Cytosolic Redox Signals

**Figure 1:**
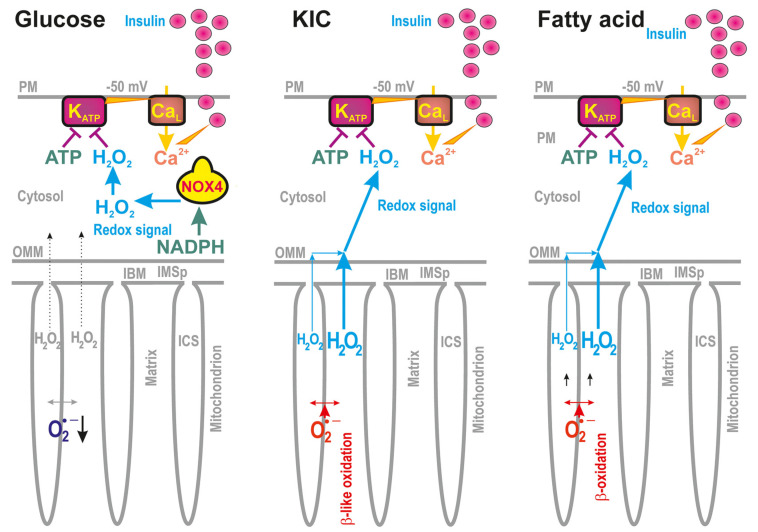
Redox signaling upon insulin secretion, stimulated either with glucose [18,19], ketoisocaproate (KIC) [18], or fatty acid [22]. Schemes show that the ATP-sensitive K^+^ channel (K_ATP_) is to be closed only when both ATP and H_2_O_2_ (redox signaling) act in synergy [18], leading to a threshold depolarization (−50 mV) of the plasma membrane and concomitant opening of the voltage-dependent Ca^2+^ channels (Ca_L_), allowing the Ca^2+^ entry and insulin granule vesicles’ exocytosis. With glucose, the pentose phosphate shuttle supplies NADPH [24] (besides so-called redox pyruvate transport shuttles, [19]) for the constitutively expressed NADPH-oxidase isoform 4 (NOX4), which provides cytosolic redox signaling [18]. With KIC, its oxidation provides both ATP and H_2_O_2_, which now originates from the mt-matrix-formed superoxide/H_2_O_2_ [18]. KIC is oxidized via a series of reactions resembling fatty acid β-oxidation (termed β-like oxidation) [13,14]. Finally, with fatty acid, even at low glucose, fatty acid β-oxidation also provides both ATP and H_2_O_2_. Again, H_2_O_2_ is of mt matrix origin and redox signaling from mitochondria to the plasma membrane (PM) has to occur. Simultaneously, H_2_O_2_ also activates mitochondrial phospholipase iPLA2γ (not shown, for simplicity), which adds a surplus of mt fatty acids for both β-oxidation and the metabotropic GPR40 receptor on PM [22]. GPR40 downstream pathways further stimulate insulin secretion.

Studying pancreatic β-cells [12,13,14,15,16,17,18,19,20,21], we have encountered situations upon the glucose-stimulated insulin secretion (GSIS) [18,19] and fatty acid- (FA)-stimulated insulin secretion (FASIS) [12,22], whereby a distinct direction of pro-oxidant vs. more reduced states occur in cytosolic vs. mitochondrial matrix compartments (Figure 1). For both, upon GSIS and FASIS, the cytosolic compartments became more oxidized via H_2_O_2_ release, whereas the mt matrix superoxide release was indicated to decline [12,13,14,18,19,22,23].

### 2.1. Distinct Redox States of Mitochondrial vs. Cytosolic Compartments

#### 2.1.1. Distinct Redox States of Mitochondrial vs. Cytosolic Compartments upon GSIS

Upon GSIS, the increased H_2_O_2_ release due to the elevated function of NADPH-oxidase 4 (NOX4) is essential for the subsequent exocytosis of insulin granule vesicles (IGVs) [18,21], while such a cytosolic redox signal co-induces the closure of the ATP-sensitive K^+^ channels (K_ATP_) on the plasma membrane together with the elevated ATP [13,14,18]. Thus, in pancreatic β-cells, the K_ATP_ closure with the aid of other non-specific calcium channels (NSCCs, such as TRMP2 channels, [17]) or Cl^−^ channels allows plasma membrane depolarization to the −50 mV threshold [15], allowing subsequent intermittent opening of Ca^2+^ channels together with counteracting voltage-dependent K^+^ channels. The resulting intermittent Ca^2+^ entry into the cytosol, detectable as cytosolic Ca^2+^ oscillations, stimulates pulsatile IGV exocytosis [13,14,15,16] (Figure 1).

Nevertheless, in the mitochondrial matrix, the opposite redox changes occur upon GSIS [19]. Due to the induction of the operation of several redox shuttles [16,19], less NADH is produced within the mt matrix, which leads to lowering mt substrate pressure (NADH_mt_/NAD^+^_mt_) onto the respiratory chain (RC) Complex I flavin site of superoxide formation (I_F_) and hence less superoxide is formed. As a result, less H_2_O_2_ is released into the mitochondrial matrix [19,23].

Among the redox shuttles verified via several reports [16,19], a pyruvate–malate shuttle is enabled via the pyruvate carboxylase (PC) reaction bypassing the regular pyruvate dehydrogenase- (PDH)-mediated entry into the Krebs cycle, while allowing the reverse reaction of the mt matrix malate dehydrogenase (MDH2), consuming NADH instead of making it. Subsequent malate export from the mt matrix benefits the cytosolic malic enzyme (ME1) which transfers malate into the pyruvate, yielding NADPH instead [19]. Notably, such additional NADPH feeds NOX4, together with the two enzymes of the pentose phosphate pathway (PPP), producing NADPH, i.e., glucose-6-phosphate dehydrogenase and 6-phosphogluconate dehydrogenase [13,14]. Metabolomics studies have determined that up to 10% of glucose flux is diverted to PPP and hence H_2_O_2_ formation via NOX4 [24]. The pyruvate–malate shuttle then represents an additional source of NADPH for NOX4.

Another experimentally verified redox shuttle is the pyruvate–isocitrate shuttle, not allowing the mt matrix isocitrate dehydrogenase isoform 3 (IDH3) to form NADH, but instead the matrix NADPH is consumed by IDH2 [19,25]. This allows the export of isocitrate from the mt matrix and reaction of cytosolic IDH1, transforming isocitrate to 2-oxoglutarate (2OG), while again forming NADPH as the surplus substrate for NOX4. Similarly, having produced typically less than three NADH in a single turn of the Krebs cycle, the RC Complex I site I_F_ forms less superoxide and the H_2_O_2_ release into the mt matrix is slowed down.

It seems that there is no problem with the above explanations or interpretations of published data. However, one may ask why the increasing cytosolic H_2_O_2_ release is not projected into the mt matrix. Are the enzyme and transport pathways of the redox shuttles so powerful that they create redox states, which are not counteracted from the cytosolic H_2_O_2_, possibly acting back to the mt matrix? Is there any “redox insulation”? How does the peroxiredoxin system [2,26] of the cytosol vs. matrix respond to these redox changes? What is the cause that enables the mt matrix compartment to be independent, despite the fact that it represents a relatively minor volume compared to the cytosolic one?

#### 2.1.2. Distinct Redox States of Mitochondrial vs. Cytosolic Compartments upon FASIS

Upon ongoing FASIS [12,22], distinct directions of H_2_O_2_ release into the cytosol vs. mt matrix are also encountered. Interestingly, there exists a functional synergy of the mt uncoupling protein 2 (UCP2) and the redox-activated mt Ca^2+^-independent phospholipase A2, isoform γ (iPLA2γ/PNPLA8). This synergy results in a mild uncoupling of the protonophoric force Δp, established on the inner mitochondrial membrane (IMM; specifically on the intracristal membranes (ICS membranes)) and hence causes a slowdown of the superoxide release into the mt matrix [22]. Indeed, the released nascent FAs, cleaved by iPLA2γ/PNPLA8 from the mt phospholipids, become cycling anionic substrates of UCP2, causing such a mild uncoupling [22,27]. The silencing/ablation of either UCP2 or iPLA2γ prevented a >50% decrease in the superoxide release into the mt matrix [22].

Nevertheless, and on the contrary, upon FASIS, at the same time, FA β-oxidation creates excessive superoxide/H_2_O_2_ (Figure 1), which is subsequently sensed by the introduced cytosolic fluorescence ROS probes in insulinoma INS-1E cells [22]. In the isolated pancreatic islets (PIs) and INS-1E cells, it even reaches the cell exterior (unpublished data). Again, how is this possible? Why does the matrix fluorescence superoxide indicator linked to the redox state shows a decrease [19,22], whereas the cytosolic [18] H_2_O_2_ probes indicate an H_2_O_2_ increase? Is there any channeling of the oxidized state from the mt membranes to the cytosol? Does the mt peroxiredoxin system [2,26] participate in such an outward-directed H_2_O_2_ flux? Is the superoxide formed within the intracristal membrane, somehow insulated from the intracristal space or the intermembrane space, so that the opposite redox changes are allowed therein?

To answer all of these questions, further research is required. To create solid theoretical predictions, we will attempt to make theoretical analyses in the further text below. They converge in two models or hypotheses. The first one is concerned with the participation of the peroxiredoxins; the second one is concerned with the activation of the mt matrix superoxide dismutase MnSOD/SOD2. The latter could explain the opposite mt matrix probe vs. the cytosolic probe responses, since paradoxically, the activation of MnSOD accelerates superoxide dismutation and this competes with the response of the superoxide-sensitive probe, such as MitoSOX. As a result, MitoSOX monitors a decreasing superoxide release rate, whereas the resulting accelerated H_2_O_2_ formation in the matrix allows a high portion of H_2_O_2_ to diffuse into the cytosol. In this way, perfect conditions for redox signaling from the mitochondria could be established.

## 3. Redox Sources vs. Redox Buffers in Mitochondria and Cytosol

### 3.1. Specificity Given by the Mitochondrion Architecture

#### Understanding Mitochondrial Compartments

**Figure 2:**
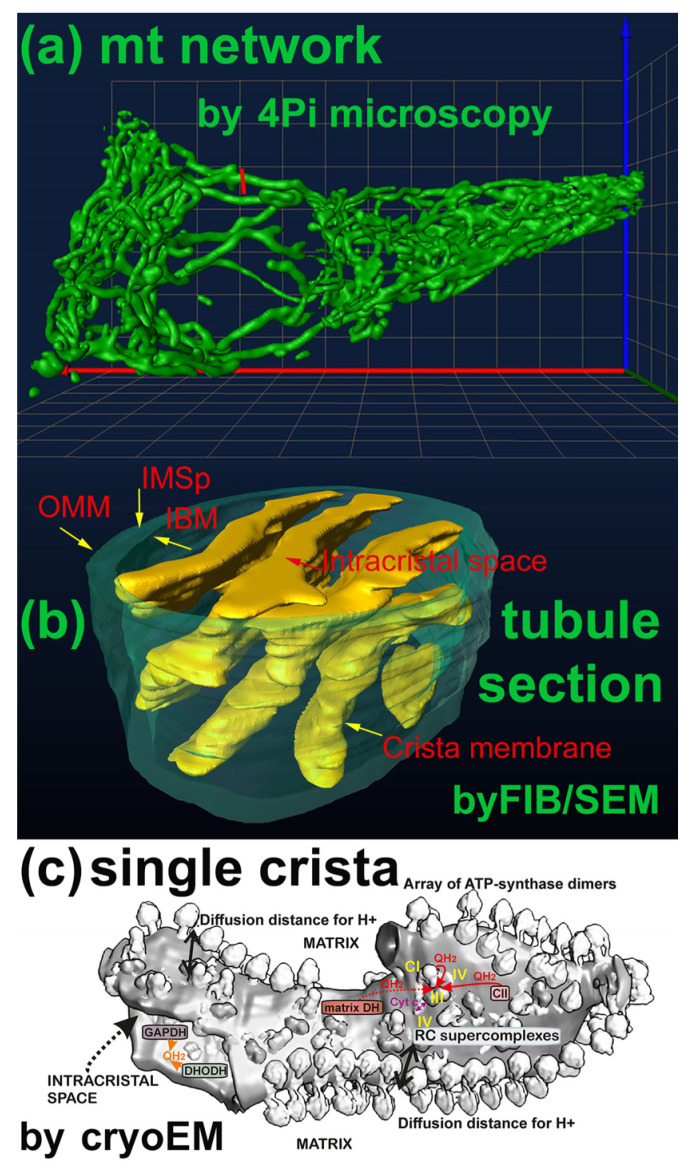
Compartments of mitochondrion. Schemes shows examples of the following: (**a**) Mitochondrial network as imaged using 4Pi microscopy, i.e., 3D high-resolution fluorescence microscopy (images, such as published in Ref. [28]). (**b**) Section of a mitochondrial network tubule (such as indicated by the red line in (**a**)), extracted from 3D FIB/SEM images (see Ref. [33]). The outer mitochondrial membrane (OMM) is highlighted by a green color, similarly to the inner boundary membrane (IBM) and the intermembrane space peripheral (IMSp) between them. Yellow color-coding is used for cristae membranes, shown together with the contained but unresolved proteins. (**c**) Image of a single crista with already-resolved ATP-synthase dimers and respiratory chain (RC) supercomplexes, adopted from Ref. [34]. Diffusion distances for ubiquinol (QH_2_) or ubiquinone (Q) are indicated by arrows, as well as the diffusion of protons between the ATP-synthase and RC supercomplexes. Matrix-faced dehydrogenases (DHs) allow QH_2_ diffusion on the same leaflet, whereas the QH_2_ diffusion between dehydrogenases facing the intracristal space (ICS) requires a flip/flop of QH_2_/Q. These are namely glycerol-3-phosphate dehydrogenase (GAPDH) and dihydroorotate dehydrogenase (DHODH).

The mitochondrion typically forms a nearly connected tubular network [28,29,30,31,32], from which small fragments are dynamically separated by fission mechanisms, while fragments join the main network via the fusion mechanism. The ultrastructural organization of mt cristae within the network (or fragments) is complex, so that at least the following compartments are recognized [31] (Figure 2). The innermost matrix compartment resembles an infinite octopus with lamellar tentacles, whereas the intracristal space (ICS) is formed by crista membrane (CM) lamellae oriented mostly perpendicularly to the nearly cylindrical outer membrane and the parallel inner boundary membrane (IBM), from which the CM are invaginated.

Around the crista outlets, which resemble a bottleneck, the MICOS-SAM complexes form so-called crista junctions (CJs), joining the IBM MICOS with the OMM SAM [31,32]. A typical crista lamella is not a regular cuboid, but an irregular structure with edges formed by the ATP-synthase dimers organized in arrays [31]. The RC supercomplexes reside on flanks of crista lamellae [34]. Cristae (crista lamellae) can shrink to provide more-efficient oxidative phosphorylation (OXPHOS) or inflate to establish less-efficient OXPHOS [33,35,36]. The thin cylindrical compartment forming the center of the OMM IBM sandwich was termed as the intermembrane space (IMS). We will refer to it as the peripheral IMS (IMSp, [31]) to distinguish IMSp from the cristae lumen space, which is in fact the ICS.

### 3.2. Key Players of Redox Equilibrium in Mitochondria and Cytosol

#### 3.2.1. Original Mitochondrial Superoxide Sources

The mitochondrion provides several sites forming superoxides. A detailed description of the mechanisms and locations of the superoxide sources can be found in published reviews, e.g., [31,37,38,39]. We should note, however, that most of the considered sites of the superoxide formation release superoxide to the mt matrix compartment. Only a part of the Complex III site III_Qo_ superoxide production is released to the ICS, as well as the superoxide produced by ICS-facing dehydrogenases (if they produce superoxide under the given conditions), such as glycerol-3-phosphate dehydrogenase (GAPDH) and dihydroorotate dehydrogenase (DHODH) [31,37,38]. Also, Complex II (succinate dehydrogenase) produces superoxide under certain conditions [31,40], namely in pathologies [31,41].

Note that the IMSp is regarded as the most oxidized compartment, due to the function of the MIA40 system oxidizing sulfhydryl pairs (located apart) of certain suitable IMS proteins into S-S bridges (disulfides) upon their import [42,43]. Moreover, CuZnSOD has been located in this compartment. It is not known whether the MIA40 system can also affect ICS [42,43]. In this review, we do not discuss the redox phenomena in the cytosol, since this would entail another review. The reader can find descriptions in references such as [4,39].

#### 3.2.2. Peroxiredoxin System

**Figure 3:**
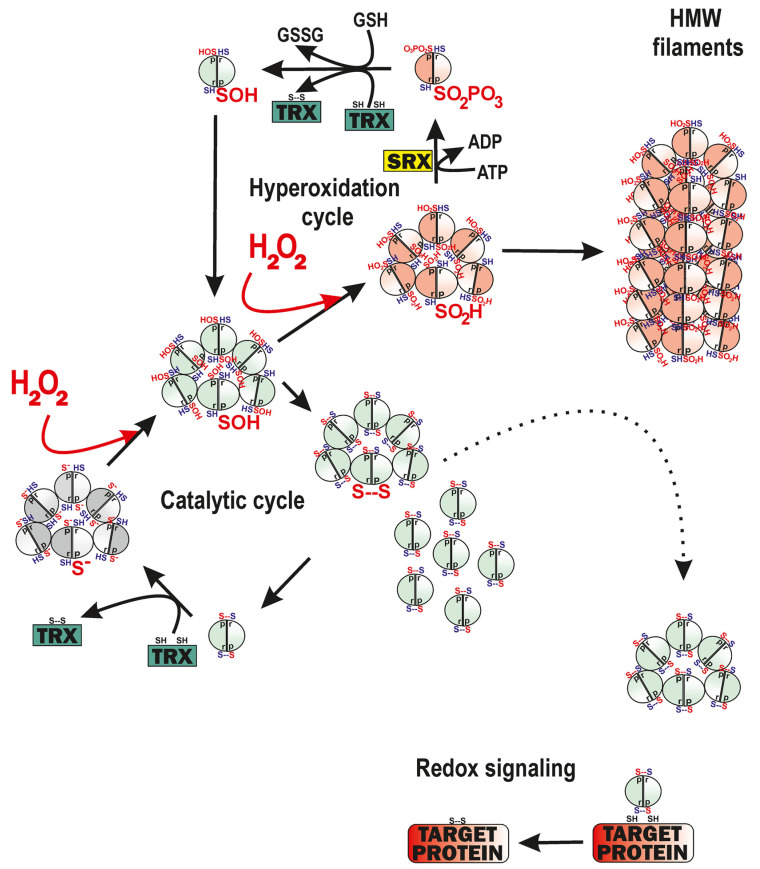
Peroxiredoxins of 2-Cys type exemplified by cycles of mt PRDX3. The catalytic cycle of the PRDX3 dodecamer emphasizes the deprotonation of the peroxidatic cysteine into S^−^ (*red*, protein part denoted as “p”), its oxidation into the sulfenyl state, and concomitant formation of S-S bonds with the resolving cysteines in the proximal subunit of the dimeric couple (*dark blue*, protein part denoted as “r”). After this, dodecamers occur in an unstable conformation, allowing splitting to dimers, which are reduced (regenerated to the original state) by the thioredoxin (TRX) plus TRXR (omitted). Upon intensive H_2_O_2_ flux, a hyperoxidation cycle starts by oxidation into sulfinyls (SO_2_H), which can stack into the HMW filaments. Sulfinyls can be reduced at the expense of the ATP, being phosphorylated during the first step via a sulfinyl reductase reaction (sulfiredoxin, SRX), followed by TRX/TRXR or glutahione (GSH)/GRX. Redox signaling can exist, when a particular target protein with proximal cysteines interacts with just the peroxiredoxin in a state with disulfides. Protein reduces the peroxiredoxin dimer, whereas being oxidized into disulfides typically alters its function.

Peroxiredoxins (PRDXs) of the 2-Cys type are decameric (dodecameric in mt) hydroperoxide reductases containing peroxidatic cysteine, C_P_. They form a toroid (doughnut-like) structure of five (six) homodimers, which can split from the toroid in an unstable disulfide conformation (Figure 3). The second resolving cysteine, C_R_, in the second homodimer subunit forms an inter-subunit disulfide bond with C_P_ upon a reaction with H_2_O_2_ [44,45,46,47,48,49,50]. At first, sulfenic acid (R-SOH) is formed via two-electron reversible oxidation. In an atypical mt peroxiredoxin, PRDX5, the intra-subunit S-S bridge, is formed, i.e., within the single subunit [44,45,46,47,48]. The PRDX ring is destabilized when disulfide bonds are formed [44,46]. Finally, the cycle is completed by the reduction of the two disulfide bonds of the homodimer, catalyzed either by a couple of thioredoxins (TRXs) plus NADPH-dependent TRX reductase (TRXR), or by glutathione (GSH)/glutaredoxin (GRX) [44,45,46,47,48] (Figure 3).

Since peroxiredoxins react with H_2_O_2_ faster than other peroxidases, such as catalases and glutathione peroxidases (GPX), they are considered as the main regulators of cytosolic H_2_O_2_ (besides NOX enzymes) and the related development of diseases with etiology involving oxidative stress. Thus, peroxiredoxins have been implicated in cancer development [49,50], as targets for cardiovascular disease [51] or neurodegenerative diseases [52], and in the β-cell defense against oxidative damage [53].

Cytosolic peroxiredoxins convey their oxidation by H_2_O_2_ to the terminal target proteins, typically phosphatases or transcription factors [44,46,54,55,56,57,58]. Thus, peroxiredoxins are literally capable of a “redox kiss” to the target protein, enabling the execution of a redox signal. A phenomenon of the so-called floodgate has been predicted to describe the shift of the oxidation from the original to distant locations [2,59,60]. It is based on the property of 2-Cys cytosolic peroxiredoxins PRDX1, PRDX2, and mtPRDX3 to form stacks of decamers/dodecamers (high-molecular-weight complexes, HMW), when sulfenyls are oxidized into higher oxidized states, i.e., to sulfinyls or even to sulfonyls [26]. HMW complexes frequently form filaments and those with sulfinyls can still be reduced by ATP-dependent sulfinyl reductase (SRX) enzymes [26,61] (Figure 3), unlike those with sulfonyls. The hyperoxidation of PRDX3 is about twice as slow than for PRDX2 [26].

According to the floodgate model, the HMWs formed in the original locations upon a sustained H_2_O_2_ flux allow oxidation in the distant loci, proximal to the target proteins, and hence enable the execution of the redox signal. HMW thus effectively withdraws PRDX molecules from the catalytic cycle; so, in their loci, PRDX oxidation cannot proceed and H_2_O_2_ is allowed to diffuse to further distances. But, PRDX-containing sulfinyls are brought back after their reduction by the SRX system. Mt SRXs were reported to act in the transfer of circadian rhythms to the mitochondrial matrix via the SRX expression intermittent with SRX degradation by LON-protease, controlled by the clock-genes in the adrenal gland, brown adipose tissue, and heart [26,62,63].

In human cells, six isoforms of PRDXs exist. PRDX1, PRDX2, and PRDX6 are localized in the cytosol and nucleus, while PRDX4 is localized in the endoplasmic reticulum [44,45,46,47,48,49,50]. The isoform PRDX3 is exclusively mitochondrial, whereas PRDX5 is located in mitochondria [64], but also in the cytosol and peroxisomes. PRDX 5 prefers lipid peroxides and peroxynitrite over H_2_O_2_. Artificial PRDX5 expression in IMSp attenuated hypoxic transcriptome reprograming as well as carcinogenesis [65,66]. PRDX6 can be recruited to mitochondria (to OMM) [67,68,69]. PRDX6 is a 1-cys-PRDX, which forms only homodimers and is unable to form disulfide bonds nor to be reduced by SRX. Its sulfenic moiety is then reduced with GSH, but not with thioredoxins. PRDX6 reduces oxidized phospholipids and also exerts Ca^2+^-independent phospholipase A2 activity.

#### 3.2.3. Other Mitochondrial Redox Buffers

Glutathione (GSH) is a major mt matrix redox buffer in numerous cells [2,4], but exerts a lower abundancy and power in certain cells, such as in pancreatic β-cells [12,13,14,15,16]. The glutathione peroxidase (GPX) family consists of five enzymes with seleno-cysteine active sites (GPX1 to 4, GPX6), utilizing GSH as a cofactor [70,71], and three other enzymes with a redox sensor role (GPX5, GPX7, and GPX8) having only cysteine residues in their active sites and modest peroxidase activity [72]. Note that the cytosolic and mitochondrial GPX1 and plasma membrane and cytosolic GPX4 are abundant in all tissues and cell types. A complete survey of the mt GPX isoform is yet to be made. Relations of GPX enzymes to the ferroptosis type of cell death have been firmly established [73]. A correlated function of the redox buffers in the mitochondrial matrix (ICS, IMSp) has to always be considered for the particular cell type and/or physiological or pathological situation.

### 3.3. Diffusion of Mitochondria-Produced H_2_O_2_ into the Cytosol and Extracellular Compartment

#### 3.3.1. Diffusion of H_2_O_2_ to the Cytosol

The primary superoxide sources, such as RC supercomplexes, can release the superoxide anion into the mt matrix compartment or into the ICS [31,37,38,39]. MnSOD, localized in the matrix, should convert the majority of superoxide into H_2_O_2_. Similarly, CuZnSOD might convert the ICS-released superoxide into H_2_O_2_ just within the ICS [74]. Note that if H_2_O_2_ was diffusing across the CM, it would reach the mt matrix within ~99% of the CM surface. Only at the proximity of CJs can the ICS-located H_2_O_2_ escape into the IMSp and subsequently via the OMM, even to the cytosol. So, considering only the ultrastructure of the mitochondrion, one can recognize how difficult it is to establish H_2_O_2_ diffusion from ICS to the cytosol. This is why, in our considerations below, we will frequently simplify a problem to a situation, only when the matrix superoxide or H_2_O_2_ release is considered. Thus, a regular diffusion of H_2_O_2_ from the matrix to the cytosol must proceed across the IBM, IMSp, and OMM.

Let us first calculate a fraction of the original mt superoxide source that might reach the cytosol. Let *J*^S^ be the original total superoxide formation rate (flux) from all mt sources. It divides into the two mentioned directions, to the ICS *J*^S^_ICS_ and to the mt matrix *J*^S^_m_:

*J*^S^ = *J*^S^_ICS_ + *J*^S^_m_ (1)

The superoxide dismutation in these compartments acts with an efficiency of ε_ICS_ and ε_m_, respectively, so that the total H_2_O_2_ release *J* into the ICS compartment will be as follows:

*J*_ICS_ = ε_ICS_ · *J*^S^_IC_ (2)

For the matrix compartment, this will be

*J*_m_ = ε_m_ · *J*^S^_m_ (3)

**Figure 4:**
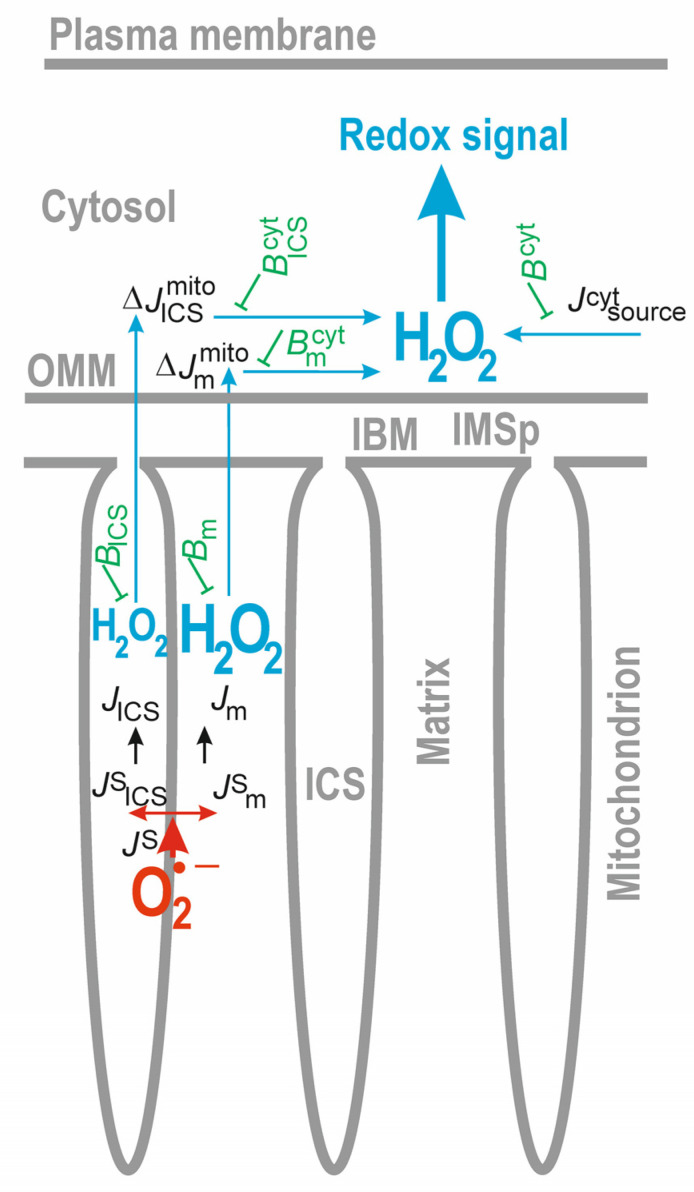
Considered fluxes and redox buffers for deriving the cytosolic redox signal (see Equation (6)).

However, these fluxes will be counteracted by the redox buffers and antioxidants. Let us consider that all matrix redox buffers/antioxidants withdraw H_2_O_2_ via the rate *B*_m_, and similarly via the rate *B*_ICS_ within the ICS. Next, any remaining and perhaps potentially escaping free H_2_O_2_ flux Δ*J*^mito^ into the cytosol (Figure 4) will be as follows:

Δ*J*^mito^_ICS_ = υ_ICS_ · (ε_ICS_ · *J*^S^_ICS_ − *B*_ICS_) (4)

and for the matrix compartment, this will be as follows:

Δ*J*^mito^_m_ = υ_m_ · (ε_m_ · *J*^S^_m_ − *B*_m_) (5)

where the only fraction υ_ICS_ or υ_m_, respectively, of free H_2_O_2_ diffuses across CJs/IMSp/OMM or IBM/IMSp/OMM, respectively.

#### 3.3.2. Convergence of Mitochondrial and Cytosolic H_2_O_2_ Fluxes

Within the cytosol, we should consider that all cytosolic H_2_O_2_ sources contribute to the cytosolic H_2_O_2_ release flux *J*^cyt^_source_, which is counteracted by the cytosol redox buffer capacity (flux) *B*^cyt^, to which we also include any H_2_O_2_ detoxification, such as the one in peroxisomes or via glutathione peroxidases. Indeed, such a cytosolic H_2_O_2_ withdrawal (redox buffer/antioxidant capacity) also counteracts the two mitochondrial H_2_O_2_ resources; so, the net cytosolic H_2_O_2_ release can be expressed as follows:

Δ*J*^cyt^ = *J*^cyt^_source_ − *B*^cyt^ + υ_m_ · (ε_m_ · *J*^S^_m_ − *B*_m_) − *B*_m_^cyt^ + υ_ICS_ · (ε_ICS_ · *J*^S^_ICS_ − *B*_ICS_) − *B*_ICS_^cyt^ (6)

Note that Δ*J*^cyt^ > 0 when

*B*^cyt^ + υ_m_·*B*_m_ + υ_ICS_·*B*_ICS_ + *B*_m_^cyt^ + *B*_ICS_^cyt^ < *J*^cyt^_source_ + υ_m_ · *J*_m_ + υ_ICS_ · *J*_ICS_ (6a)

This refers to when the H_2_O_2_/superoxide sources overcome the redox buffering/antioxidant capacities in all of the compartments involved, i.e., Σ*B*. If one would attempt to estimate the extracellular H_2_O_2_ release, the fraction of υ_PM_, i.e., the fraction of *J*^cyt^, which is able to cross the plasma membrane (PM), must be considered. Hence, it is valid that

Δ*J*^extracellular^ = Δ*J*^cyt^ · υ_PM_ (7)

In the case where there are no intense cytosolic H_2_O_2_ sources, the relationship (6a) turns into the following:

Σ*B* < υ_m_ · *J*_m_ + υ_ICS_ · *J*_ICS_ (7a)

### 3.4. Distinct Redox State Changes in Cytosolic vs. Matrix Compartments

#### 3.4.1. When Is This Possible?

Let us analyze whether the above simplified equations allow us to distinguish between redox state changes in the cytosolic vs. matrix compartments. We consider the cytosolic change as that one in the peri-plasma membrane locations or the one projecting up to the extracellular space. Let us also consider for simplification that ε_ICS_ · *J*^S^_ICS_ = *B*_ICS_, so that the last term in Equation (6) is zero. Let us further approximate that υ_m_ = 1. As a result, we obtain a simplified relationship, as follows:

Δ*J*^cyt^ = *J*^cyt^_source_ − *B*^cyt^ + ε_m_ · *J*^S^_m_ − *B*_m_ − *B*_m_^cyt^ (8)

This Δ*J*^cyt^ flux of H_2_O_2_ is positive when

*J*^cyt^_source_ > *B*^cyt^ + *B*_m_ + *B*_m_^cyt^ − *J*_m_ (8a)

We may conclude that when the mitochondrial source decreases (upon ongoing antioxidant mechanisms), there could still exist a pro-oxidant H_2_O_2_ release in the cytosol, if its source exceeds the redox buffering capacities plus a drop in the mitochondrial source. *Vice versa*, when there is a negligible cytosolic H_2_O_2_ source, the mitochondrial one must overcome all of the redox buffers/antioxidants in the compartments. One can observe that the conditions of Equation (8a) exist upon GSIS, when the *J*^cyt^_source_ is represented by the NOX4, whereas the redox pyruvate shuttles provide a decrease in *J*_m_ with time. When H_2_O_2_ efflux from peroxisomes is possible [75], *J*^cyt^_source_ should contain such a component.

#### 3.4.2. Accelerated MnSOD Activity

One can also consider that the MnSOD activity could be increased upon certain stimuli. To solve this case, we should consider and experimentally evaluate superoxide release and H_2_O_2_ production, diffusion, and release to the matrix vs. cytosolic compartments separately. We must therefore calculate the remaining superoxide flux Δ*J*^S^_m_ as the difference between sources and consumption. The major part of consumption is the dismutation by MnSOD, expressed by the term *J*^SOD^_m_. Moreover, phenomena diminishing the original superoxide formation, such as uncoupling or interaction with mt constituents, etc., can be described by another term, introducing the consumption of the superoxide by a flux *J*^S^_m_^consuming^. The balance of the matrix superoxide is then expressed as follows:

Δ*J*^S^_m_ = *J*^S^_m_ − *J*^SOD^_m_ − *J*^S^_m_^consuming^ (9)

When, e.g., *J*^S^_m_^consuming^ plus *J*^SOD^_m_ with time are greater than the original superoxide source *J*^S^_m_, the superoxide release with time decreases. Experimentally, probes such as MitoSOX Red should monitor the decreasing rates of the superoxide release into the mitochondrial matrix in such a case [19]. Nevertheless, upon MnSOD activation (denoted by Index *2*, apart from the original state denoted by Index *1*) not only does *J*^SOD^_m_(*2*) increase, but the resulting H_2_O_2_ matrix release also increases. Therefore, substituting Equation (9) into Equation (5), we can obtain

Δ*J*^mito^_m_(*1*) = υ_m_ · {ε_m_ · [*J*^S^_m_(*1*) − *J*^SOD^_m_(*1*) − *J*^S^_m_^consuming^(*1*)] − *B*_m_} (10)

Δ*J*^mito^_m_(*2*) = υ_m_ · {ε_m_ · [*J*^S^_m_(*2*) − *J*^SOD^_m_(*2*) − *J*^S^_m_^consuming^(*2*)] − *B*_m_} (10a)

The net change in Situations (*2*) − (*1*) when *J*^S^_m_(*1*) = *J*^S^_m_(*2*) and *J*^S^_m_^consuming^(*1*) = *J*^S^_m_^consuming^(*2*) will be as follows:

Δ*J*^mito^_m_(*2* − *1*) = υ_m_ · ε_m_ · [*J*^SOD^_m_(*1*) − *J*^SOD^_m_(*2*)] (10b)

Since the term *J*^SOD^_m_(*2*) is larger than *J*^SOD^_m_(*1*), the resulting change in H_2_O_2_ release penetrating into the cytosol will be positive and non-zero. We conclude that if under a certain stimulus MnSOD activity increases, one can experimentally indicate the diminishing superoxide with time in the matrix, compared with the increasing H_2_O_2_ with time in the cytosol.

#### 3.4.3. Examples of MnSOD Regulation

Acute switch-on of MnSOD activity can only be realized via fast post-translational modifications (PTMs). One of the frequently documented PTMs is the NAD^+^-dependent sirtuin-3 (SIRT3)-mediated deacetylation of MnSOD [76,77,78]. There could be a conceptual problem with this, since it is not a large population of enzymes in the matrix that is acetylated/deacetylated, but only a fraction. Nevertheless, in our cases of the intramitochondrial redox signal or weak redox signaling to the cytosol, such a fraction can still produce a sufficient H_2_O_2_ bolus. Concerning MnSOD regulations within an hour time frame or chronic regulations, subtle changes accumulated during the time can also be effective [79,80,81,82,83].

### 3.5. Taking into Account the ICS Volume Changes

#### The ICS Volume Changes upon Substrate Variations

Nature and mitochondrion are more complex for the simplified description, as discussed above. Upon a respiration substrate increase, cristae narrowing has been observed [33], as well as preceding cristae inflation upon hypoxic cell adaptation [35]. We have also encountered cristae narrowing upon GSIS [36]. Let us consider the relative rates before glucose to be Δ*J0*^mito^_ICS_ = 50 and Δ*J0*^mito^_m_ = 150 (relative magnitudes based on published data of Ref. [22]), while taking the ICS volume *V0*_ICS_ to be twice the volume *V*_ICS_ established after shrinkage and resulting from a high glucose addition (upon GSIS). *V0*_ICS_ = 2 *V*_ICS_. We may also reasonably expect and define that *V*_m_ = 3*V*_ICS_. Since the OMM/IBM volume is constant, we can consider that the matrix volume inflates by the same volume through which the ICS volume shrinks. Hence, *V*0_m_ = (2/3) · *V*_m_.

Considering volumes, one can introduce flux *per* volume as a flux density *ρ* in a given compartment. As expressed in our relative units prior to GSIS, this will be *ρ0*_ICS_ = 50/2*V*_ICS_, which gives 75/*V*_m_. For the matrix, *ρ0*_m_ = 225/*V*_m_. After the volume changes, *ρ*_ICS_ = 50/*V*_ICS_, which gives 150/*V*_m_ and *ρ*_m_ = 100/*V*_m_. Simply, the cristae narrowing to a half volume causes the effective doubling of the ICS H_2_O_2_ flux density, whereas it reduces the matrix flux density to about 2/3. These theoretical considerations match the observed values [22].

These considerations and exemplar cases also call for the necessity of measurements of redox states in the ICS and matrix compartments separately. While the MitoSOX Red, MitoB [19], and mitochondria-matrix-addressed HyPer probes monitored redox changes in the matrix compartments, the ICS redox changes were only scarcely evaluated.

## 4. Floodgate Effect of Peroxiredoxins May Promote Redox Signaling

### 4.1. Intramitochondrial Redox Signaling Promoted by PRDX3

#### 4.1.1. The Simplest Model

In the simplified model, we consider that the hyperoxidation cycle of PRDX3 is too slow, relative to the catalytic cycle (Figure 3). Consequently, we do not need to calculate the rates for the hyperoxidation cycle and consider them as negligible. We will also neglect the splitting into homodimers. In such a case, the total amount of PRDX3 dodecamers [PRDX3_12_] only consists of two major portions. The first fraction [PRDX3_12_]_SOH_ is only oxidized into the first, i.e., sulfenyl state, and remains within the catalytic cycle (Figure 3). The second fraction [PRDX3_12_]_SO2H +SO3H_ is withdrawn from the catalytic cycle, forms HMW structures, and reacts with H_2_O_2_ only when converting the remaining sulfinyls (R-SO_2_H) into sulfonyls (R-SO_3_H). Using Equation (5), we can neglect other redox buffering power and take the quantity *B^SOH^*_m_ as the rate of H_2_O_2_ being withdrawn by the PRDX3 catalytic cycle plus the diminishing rate of oxidation into sulfonyls, *B^SO3H^*_m_. However, note that *B^SOH^*_m_ decreases with time at the expense of the increasing *B^SO2H^*_m_. The remaining mt matrix H_2_O_2_ flux will, in this case, be as follows:

Δ*J*^mito^_m_/υ_m_ = ε_m_ · *J*^S^_m_ − *B^SOH^*_m_ − *B^SO2H^*_m_ − *B^SO3H^*_m_ (11)

where at the decrease in the catalytic cycle at a later time, (*2*) will diminish the H_2_O_2_ withdrawal by the rate *B^SOH^*_m_(*2*), so that

*B^SOH^*_m_(*1*) − *B^SOH^*_m_(*2*) = *B^SO2H^*_m_(*2*) − *B^SO2H^*_m_(*1*) (12)

and the difference between the net late and the net initial H_2_O_2_ flux at υ_m_ = 1 will be as follows:

Δ*J*^mito^_m_(*2*) − Δ*J*^mito^_m_(*1*) = *B^SO3H^*_m_(*1*) − *B^SO3H^*_m_(*2*) (13)

Since *B^SO3H^*_m_ is decreasing and approaches zero with time, the later H_2_O_2_ flux at a later time (*2*), Δ*J*^mito^_m_(*2*), will be greater than the initial Δ*J*^mito^_m_(*1*). This consideration stands for a proof of concept for PRDX3 facilitation of the mitochondrial redox signaling.

Moreover, Equations (11)–(13) describe a steady state and do not consider a faster diffusion of H_2_O_2_ relative to the diffusion of PRDX3 dodecamers. This difference probably also stands for another cause of the floodgate model mechanism. Within the ~20 nm thin compartments of the mt matrix between the cristae lamellae, H_2_O_2_ produced by MnSOD molecules distant from the IBM/IMSp/OMM sandwich can escape from the local consumption, not only because of the local floodgate mechanism, being unable to further oxidize HMW structures, but also due to the lack of reduced PRDX dodecamers in the same *loci*. The escape means diffusion up to the IBM and across the IBM/IMSp/OMM, up to the cytosol. However, experiments with a complete ablation of PRDX3 cannot directly evidence the floodgate mechanism, since the total matrix redox buffer capacity is reduced and H_2_O_2_ diffusion is thus facilitated via the PRDX3 ablation, per se.

We have to also note that hyperoxidized PRDX3 was not detected in the whole pancreases [26]. This does not mean that in a few percent of pancreas mass, represented by β-cells, there is no hyperoxidized PRDX3. An intriguing hypothesis that similar circadian control of the matrix redox buffering capacity, such as in the adrenal gland, also exists in β-cells is still speculative. Nevertheless, the acetylation of PRDX3 was found to facilitate its hyperoxidation in INS-1E cells and in human cultured β-cells at 30 mM glucose [84].

#### 4.1.2. The Model Involving the Hyperoxidation Cycle of PRDX3

Introducing a more realistic model also considering the hyperoxidation cycle of PRDX3, one should consider that within the given time frame, the sulfinic peroxidation cycle increases the [PRDX3_12_]_SOH_ fraction, and so it does not decline as quickly as in the simplest model, as described above. The surplus of *B^SOH^*_m_(*2*) will be given by the differences in rates of the SRX hyperoxidation cycle *B^SRX^*_m_(*2*) − *B^SRX^*_m_(*1*). Hence, one can consider a higher intensity of mt redox signaling with lower activity of the SRX reaction reducing sulfinyls. This may occur, for example, with a faster rate of LON-protease-facilitated SRX degradation [26].

## 5. Pitfalls of Redox Signaling Indications with Fluorescence Probes

### 5.1. Fluorescence Monitoring of H_2_O_2_ in Cytosolic and Matrix Compartments

#### 5.1.1. Understanding Redox Buffering via Redox Probes

Unfortunately, even the most specific fluorescence probes perturb the redox system in the compartment where they are located. Hence, one has to consider them as a surplus of the counteracting redox buffering. We can simplify such a situation via the notion that introducing the fluorescent probes makes redox signaling more difficult. For probes required to be expressed, different expression levels, namely the mild ones, should be adjusted, as well as the concentrations of the concentrating probes (e.g., the MitoSOX superoxide sensor as an exemplar probe with the TPP moiety, allowing penetration to the mt matrix).

Equation (8) can be rewritten to express a contribution of the mt matrix fluorescence probe, as follows:

*J*^cyt^ = *J*^cyt^_source_ − *B*^cyt^ + ε_m_ · *J*^S^_m_ − *B*_m_ − *B*^probe^_m_ − *B*_m_^cyt^ (14)

Similarly, Equation (8) can be rewritten to express a contribution of the cytosolic fluorescence probe:

*J*^cyt^ = *J*^cyt^_source_ − *B*^cyt^ − *B*^cyt probe^ + ε_m_ · *J*^S^_m_ − *B*_m_ − *B*_m_^cyt^ (15)

One can foresee the decreases in the estimated mitochondrial and cytosolic fluxes according to the relative buffering capacity of the probe vs. the buffering capacity of its environment.

#### 5.1.2. Is the Sensitivity of Redox Probes High Enough to Be Able to Compete with Redox Buffers?

Even the recently developed H_2_O_2_-selective fluorescence probes, such as HyPer7, do not always match the required sensitivity for the indication of the net local Δ*J*^cyt^, i.e., local H_2_O_2_ surplus concentration changes with time in the observed loci [85,86,87,88]. The reason for this is the competition of the redox probe with the local redox buffers, such as with the peroxiredoxin system [85]. Matching the sensitivity to successfully indicate any surplus of local H_2_O_2_ is naturally different in the different cell types and/or situations.

We have successfully monitored redox signals upon insulin secretion in model rat pancreatic β-cells, INS-1E cells, and isolated pancreatic islets [18], and identified its origin via NOX4 [18] or from mitochondria—with the latter being in the case of fatty acid- [22] and ketoisocaproate-stimulated insulin secretion [18]. One could ascribe the ability of mt-addressed or cytosolic fluorescence probes used to the relatively low redox buffering capacity of pancreatic β-cells. Indeed, attempting HyPer7 monitoring of cytosolic H_2_O_2_ using confocal microscopy, we encountered subtle increases in the 488 nm vs. 405 nm fluorescence ratios after glucose addition to INS-1E cells at the margins of the probe sensitivity (Engstová, H. and P.J.; unpublished). On the contrary, the artificially induced superoxide/H_2_O_2_ in *C. elegans* using Supernova (an optogenetic superoxide source, [89]) yielded sufficient increases in HyPer7 488 nm vs. 405 nm fluorescence ratios to monitor H_2_O_2_ elevations [90].

Recently, HyPer7 monitoring in HEK293 cells and HeLa cells has reflected a heterogeneity between individual cells [86]. Authors have reported mitochondria-released H_2_O_2_ on the surface of the mt tubules (OMM) and in the bulk cytosol, but not in the proximity of the plasma membrane or in the nucleus. The observed H_2_O_2_ gradient was found to be under control by cytosolic peroxiredoxins and variations in the cytosolic thioredoxin reductase levels [86]. These authors accepted a possibility of H_2_O_2_-mediated redox signaling under specific metabolic conditions.

#### 5.1.3. Pitfalls in Calibration of Redox Probes

Another problem with cytosolic or mitochondrial redox probes is their calibration. Of course, attempts to calibrate with externally added H_2_O_2_ face the problem that H_2_O_2_ diffusion into the cytosol is counteracted by the cell redox buffer systems, and therefore the real cytosolic H_2_O_2_ concentrations are not matched. An alternative can be viewed in the controlled H_2_O_2_ delivery, such as introduced with an ectopic expression of D-amino acid oxidase (DAAO), which catalyzes H_2_O_2_ formation using D-amino acids [91]. In this case, the DAAO-produced amount of H_2_O_2_ can be calibrated from the concomitant oxygen consumption [91].

#### 5.1.4. Extracellular H_2_O_2_ Indications with Amplex Red—Is This the Solution to the Problem?

Solving the redox buffer problem for cytosolic redox probes might appear to be simple, lying in the use of the external probes, to be calibrated in the absence of redox buffers. Indeed, Amplex Red has been reported to indicate extracellular H_2_O_2_ originating from mitochondria [92,93,94,95]. For example, in C2C12 myoblasts, contributions of distinct sites of mt superoxide formation have been reported [94]. The RC Complex I site I_Q_ accounted for 12% (25% after differentiation into myotubes) of the total mt of superoxide/H_2_O_2_ formation, while the Complex III site III_Qo_ accounted for 30%. In cultured cells, approximately 60% of the total cell H_2_O_2_ flux, surveyed extracellularly, was ascribed to NADPH oxidases, while 30% was ascribed to mitochondria [91]. Possible obstacles with the Amplex Red method may come from the requirement of the horseradish peroxidase to catalyze Amplex Red conversion to resorufin.

### 5.2. Fluorescence Monitoring of Matrix Superoxide

The MitoSOX-Red-based confocal microscopy semi-quantification of the mitochondrial matrix superoxide is not perfect, but it is still a usable method to monitor time-lapsed mt superoxide release [96]. Due to the ability of MitoSOX Red to intercalate into mtDNA, the probe cannot leak out from the mt matrix with decreasing mt membrane potential ([19,96,97] and Supplemental Information for Ref. [19]), and hence, MitoSOX Red can also monitor situations whereby such decreases occur. MitoSOX Red fluorescence elevation could be a genuine measure of the increased superoxide release into the mt matrix, if surveyed within time intervals when the effects of MitoSOX on mtDNA are still not manifested.

### 5.3. Guidelines for Measuring ROS and Oxidative Damage

Recommended guidelines for the use and interpretation of measurements with various ROS probes and for investigations into redox states can be found in excellent published reviews [98,99,100,101,102]. Table 1 summarizes the considered advantages or disadvantages of each probe or approach.

Even more caution has to be paid when analyses of clinical samples are conducted [98]. One has to not only consider the ROS sources and their scavenging and antioxidant mechanisms, but all lipid, protein, and DNA (mtDNA) modifications and their consequences in altered autophagy (both elevated as well as decreased autophagy can be pathological) and mitochondria-specific autophagy, i.e., mitophagy. Moreover, responses by cells should be taken into account, such as the ER stress or unfolded protein response and mitochondrial unfolded protein response. The initiation or execution of various types of cell death underlie these phenomena due to their striking consequences and changes, including mt network morphology [32] and cristae ultrastructural changes [31]. Concerning mitochondria as a central metabolic and information hub [3], controlling the majority of the above-mentioned phenomena, mitochondrial biogenesis and protein turnover can delay certain pathological situations, but can serve as specific markers of the abnormal situation.

## 6. Future Perspectives

All of the above knowledge is a substantial part of modern molecular biomedicine. Further investigations into and quantifications of mitochondrial redox signals are required. Here, a small attempt is made to illustrate certain examples, where the impaired mitochondrial redox signaling might be part of the disease etiology (Table 2). It is not distinguished by timing, concerning whether the involved redox signals are acute or proceed slowly as redox regulations with concomitant gene expression changes. In a few cases, such an impairment is concerned with either the blockage or overwhelming of the repeatable acute redox signals; in most cases, exemplar redox signaling controlling gene expression is mentioned. Also, a few examples are listed for cytosolic redox signals, such as those initiated via NADPH oxidases.

Thus, mt redox signals are implicated in aging [116,117,118], as well as in the development of cancer and metastases [119,120,121,122,123]. Cytosolic redox signals/gene regulations are hypothetically involved in type 2 diabetes changes in pancreatic β-cells. The housekeeping and maintenance of the insulin gene and specific genes for pancreatic β-cells [124] were hypothetically ascribed to repeatable NADPH-oxidase-4-mediated redox signals determining glucose-stimulated insulin secretion [18]. Logically, the attenuation or blockage of these redox signals disrupt the correct housekeeping of the most important β-cell-specific transcripts. In the long term, this leads to a dedifferentiation of β-cells or their transdifferentiation into other islet cell types (e.g., α-cells and δ-cells). Redox signals or regulations of mt origin are also implicated in insulin resistance development in peripheral tissues [125,126]. Other listed examples include heart [127,128,129] and endothelial pathology [130,131,132,133,134], pulmonary hypertension [135], sepsis [136], skeletal muscle injuries (here, mt ROS signal for repair mechanisms, [137,138]), and NLR family pyrin domain-containing 3 (NLRP3)-related inflammatory states [139,140,141]. This list is illustrative and incomplete.

## Figures and Tables

**Table 1:** Probes for monitoring of superoxide, H_2_O_2._

Probe	Advantage/Disadvantage	References
O_2_^•−^, EPR, spin trapping	Rather complex snapshots ^1^	[103,104,105]
O_2_^•−^ *fluorescence monitoring*		
hydroethidine	LC-MS to distinct E^+^ vs. 2HE^+^	[106]
NeoD	No DNA intercalation	[107]
MitoSOX	Time course ^2^ vs. background separates 2HE^+^	[19,22,96,97]
MitoNeoD	No DNA intercalation ^3^	[107]
H_2_O_2_ *detection*		
Boronate- and borinate-probes	Boronates are insensitive	[108,109,110,111]
MitoB	snapshots ^1^	[19,112]
H_2_O_2_ *fluorescence monitoring*		
HyPer7	Still insens. for redox signals ^4^	[85,86,87,90]
MitoHyPer		[19,23,85,86,87,90]
Orp1		[113]
TSA2		[114]
TPX1		[115]
Amplex UltraRed with HRP	Extracellular monitoring	[37,38,40,94]
*Non-specific ROS fluorescence monitoring*		
2′,7′dichlorodihydrofluorescein	Downstream H_2_O_2_ products ^5^	[18,98]

^1^ Time resolving below minutes is not realistic; ^2^ see Ref. [19] on how to eliminate ethidium E^+^ background insensitive to superoxide. ^3^ But, mtDNA intercalation of MitoSOX is an advantage, since the probe is not redistributed upon potential changes. ^4^ See Refs. [86,87]. ^5^ Reacts with several ROS, and not with H_2_O_2_ but with products of the H_2_O_2_ reaction with redox-active metals, heme proteins, or peroxidase; see Ref. [98].

**Table 2:** Pathologies with involvement of impaired redox signaling.

Disease/Pathological State	Signal Impaired	References
Aging	Pleiotropic	[116]
Astrocyte-related	mt H_2_O_2_	[117]
Atherosclerosis	mt H_2_O_2_	[118]
Cancer	Complex II—ROS	[119]
Colon cancer	mt H_2_O_2_	[120,121]
Cancer—melanoma	mt H_2_O_2_	[122]
Cancer—pancreatic	mt H_2_O_2_	[123]
Diabetes—type 2/*Ins* gene maintenance, β-cell dedifferentiation	Post-prandial NOX4-H_2_O_2_ signals ^1^	[18,124]
Diabetes—type 2, insulin resistance	Pleiotropic	[125,126]
Heart—cardiomyopathy	mt H_2_O_2_	[127,128]
Heart—early diabetic	Complex I—ROS	[129]
Hypertension—endothelial dysfunction	Complex III—ROS	[130,131]
Hypertens—endothelial	mt ROS—induced NOX	[132,133,134]
Pulmonary hypertension	mt H_2_O_2_	[135]
Sepsis, NLRP3-related	mt H_2_O_2_	[136]
Skeletal muscle injury repair	mt H_2_O_2_	[137,138]
Various NLRP3-related	mt H_2_O_2_	[139,140]
Various NLRP3-related	NOX4, fatty acid β-oxidation	[141]

^1^ Still hypothetical.
